# Association between urinary sodium-to-potassium ratio and BNP in a general population without antihypertensive treatment and cardiovascular diseases: the Ohasama study

**DOI:** 10.1038/s41440-025-02266-0

**Published:** 2025-06-27

**Authors:** Tomoko Muroya, Michihiro Satoh, Hirohito Metoki, Shingo Nakayama, Takuo Hirose, Takahisa Murakami, Yukako Tatsumi, Ryusuke Inoue, Megumi Tsubota-Utsugi, Azusa Hara, Mana Kogure, Naoki Nakaya, Kei Asayama, Kyoko Nomura, Masahiro Kikuya, Atsushi Hozawa, Takayoshi Ohkubo

**Affiliations:** 1 Division of Public Health, Hygiene and Epidemiology, Tohoku Medical and Pharmaceutical University, Sendai, Japan; 2 Division of Internal Medicine, Izumi Hospital, Sendai, Japan; 3 Nanatsumori Family Clinic, Miyagi, Japan; 4 Department of Preventive Medicine and Epidemiology, Tohoku Medical Megabank Organization, Tohoku University, Sendai, Japan; 5 Department of Pharmacy, Tohoku Medical and Pharmaceutical University Hospital, Sendai, Japan; 6 Tohoku Institute for Management of Blood Pressure, Sendai, Japan; 7 Division of Nephrology and Endocrinology, Faculty of Medicine, Tohoku Medical and Pharmaceutical University, Sendai, Japan; 8 Division of Integrative Renal Replacement Therapy, Faculty of Medicine, Tohoku Medical and Pharmaceutical University, Sendai, Japan; 9 Division of Aging and Geriatric Dentistry, Department of Rehabilitation Dentistry, Tohoku University Graduate School of Dentistry, Sendai, Japan; 10 Department of Hygiene and Public Health, Teikyo University School of Medicine, Tokyo, Japan; 11 Department of Medical Information Technology Center, Tohoku University Hospital, Sendai, Japan; 12 Laboratory of Social Pharmacy and Epidemiology, Showa Pharmaceutical University, Tokyo, Japan; 13 Department of Environmental Health Science and Public Health, Akita University Graduate School of Medicine, Akita, Japan; 14 Division of Epidemiology, School of Public Health, Tohoku University Graduate School of Medicine, Sendai, Japan

**Keywords:** Urinary sodium-to-potassium ratio, Natriuretic Peptide, Brain, Epidemiology, Urine, Heart Failure

## Abstract

The urinary sodium-to-potassium (Na/K) ratio is associated with blood pressure (BP) and cardiovascular risk. We examined the association between the urinary Na/K ratio and brain natriuretic peptide (BNP), a biomarker indicative of cardiac stress levels within the general population. This cross-sectional study included 436 participants (mean age: 65.4 ± 6.9 years; 73.2% women) without antihypertensive medications or cardiovascular diseases (including atrial fibrillation) from the Ohasama Study. The urinary Na/K ratio was calculated using casual daytime spot urine samples. Analyses of covariance and multiple linear and Poisson regression models were conducted. The median BNP value was 18.6 pg/mL (interquartile range: 11.4–31.2 pg/mL). Participants in the first (≤2.19), second (2.19–3.27), and third (≥3.28) tertiles of the urinary Na/K ratio had adjusted mean natural log-transformed (ln)BNP of 2.74, 2.88, and 3.06 (converted BNP values: 15.50, 17.81, and 21.37 pg/mL), respectively, after adjusting for covariates including estimated glomerular filtration rate, home systolic BP, and Sokolow–Lyon voltage (*P* for trend = 0.0005). The adjusted prevalence ratios (95% confidence intervals) for BNP ≥35 pg/mL were 1.27 (0.76–2.14) and 2.24 (1.35–3.72) in the second and third tertiles, respectively, compared with the lowest tertile. The highest standardized regression coefficient for lnBNP was observed for the urinary Na/K ratio ( | 0.24 | ), surpassing estimated 24-h urinary sodium ( | 0.16 | ) or potassium ( | 0.09 | ) excretion. In conclusion, urinary Na/K ratio was associated with elevated BNP levels in individuals without antihypertensive treatment and cardiovascular disease history. This urinary marker may be valuable for early prevention of organ damage and cardiac burden.

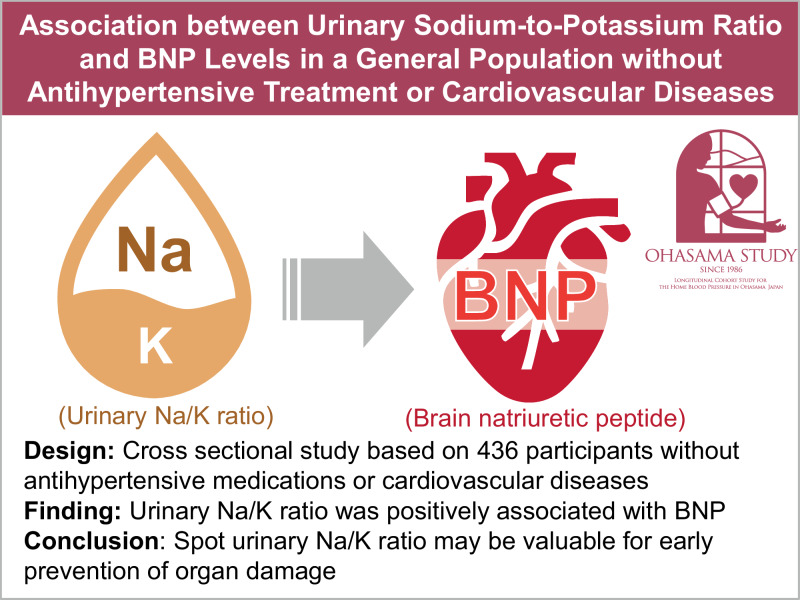

## Introduction

The urinary sodium-to-potassium (Na/K) ratio is widely recognized as a risk factor for hypertension [1–6]. Recent studies have shown that the urinary Na/K ratio is more strongly associated with blood pressure (BP) levels and cardiovascular disease risk than sodium (Na) or potassium (K) measurements alone [1, 7–12]. Controlling Na intake and increasing K intake, achieved through increased consumption of vegetables and fruits alongside salt reduction, has been widely recommended to prevent hypertension [13]. In recent years, the prevalence of heart failure has increased, raising concerns about a potential global heart failure pandemic [14, 15]. Maintaining appropriate lifestyle habits, including reduced salt intake, is important for preventing heart failure onset and progression.

Human brain natriuretic peptide (BNP) is primarily secreted by cardiac ventricles in response to increased wall tension and volume overload. Thus, it has been widely used as a clinical biomarker of heart failure [16–18]. The association between salt intake and BNP is independent of BP [19]. Moreover, changes in salt intake have been linked to changes in BNP levels [20, 21]. However, limited information exists on the association between BNP levels and the urinary Na/K ratio rather than salt intake alone. The urinary Na/K ratio is associated with an increased risk of cardiovascular disease [4, 22]. Moreover, when compared to urinary Na excretion or K excretion alone, the urinary Na/K ratio demonstrates a stronger association with BP levels [7, 23]. Given that high BP is a well-established risk factor for heart failure [16–18], it is plausible that a high urinary Na/K ratio could contribute to elevated BNP levels.

This study aimed to examine the association between the urinary Na/K ratio and BNP levels in the general population. In addition, we evaluated the potential mediating effects of BP and electrocardiographic left ventricular hypertrophy on this association [20, 24].

Point of view

- **Clinical relevance:** The urinary sodium-to-potassium (Na/K) ratio provides a practical marker for identifying individuals with high brain natriuretic peptide (BNP) before overt cardiovascular disease develops and before antihypertensive treatment is initiated.
- **Future direction:** Prospective studies incorporating detailed dietary data and echocardiographic findings are needed to establish whether interventions reducing urinary Na/K ratio can effectively lower BNP levels and prevent heart failure.
- **Consideration for the Asian population:** Given the traditionally high-sodium diets in Asian countries, the urinary Na/K ratio can be valuable for monitoring and guiding dietary modifications in Asian populations.

## Methods

### Study design

This cross-sectional study was conducted as part of the Ohasama Study, an ongoing project in Ohasama Town, Japan. The socioeconomic and demographic characteristics of this region have been previously described [23, 25–29]. The study adhered to the tenets of the Declaration of Helsinki and was approved by the Institutional Review Boards of Teikyo University (approval number: 16-075-8) and Tohoku Medical and Pharmaceutical University (approval number: 2022-005 [2019-0-006]).

### Participants

**Fig. 1:**
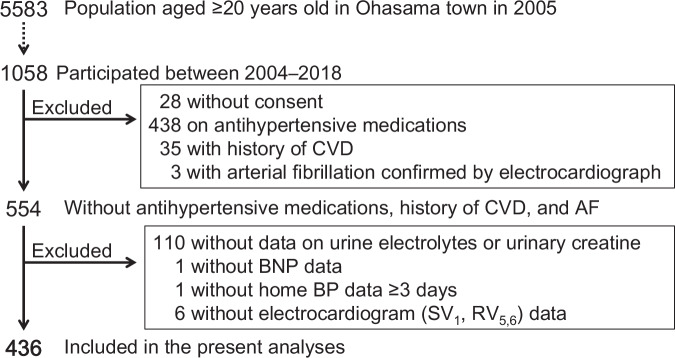
Flow diagram of participant selection. CVD cardiovascular disease, AF atrial fibrillation

Data were obtained from 1058 residents who participated in examinations conducted between 2004 and 2018. From this cohort, we selected 554 participants who were not taking antihypertensive medications and had no history of cardiovascular disease or atrial fibrillation (confirmed by electrocardiography). Of these, 118 participants with missing or incomplete data were excluded. Finally, 436 participants were included in the final analysis (Fig. 1).

### Urinary Na/K ratio data

Casual spot urine samples were collected on the day of the examination at the study center. The urinary Na/K ratio was calculated by dividing the Na concentration (mmol/L) by the K concentration (mmol/L) in the spot urine sample. As a secondary target exposure factor for comparison with urinary Na/K ratio, estimated 24-h urinary Na and K excretion (mmol/day) were calculated using Tanaka’s formulas as follows: the predicted values of 24-h urinary creatinine (Cre) excretion (Pcr) = body weight (kg) × 14.89 + height (cm) × 16.14 - age × 2.04–2244.45, estimated 24-h urinary Na excretion = 21.98 × (spot urinary Na excretion / [spot urinary Cre x 10] × Pcr) ^0.392^, estimated 24-h urinary K excretion = 7.59 × (spot urinary K excretion / [spot urinary Cre × 10] × Pcr) ^0.431^ [30]. In addition, spot urinary Na/Cre and K/Cre ratios (mmol/g Cre) were calculated by dividing spot urinary Na and K concentrations by urinary Cre concentration as simple indices of Na and K excretion.

### BNP and covariates

Blood samples were collected by trained nurses on the examination day, and serum BNP levels were measured using a chemiluminescent enzyme immunoassay.

Trained staff recorded participant anthropometric measurements and collected the following data through questionnaires and interviews: medical history (including history of cardiovascular diseases), medication use (such as antihypertensive drugs), and lifestyle factors (including smoking and alcohol consumption). Dyslipidemia was defined as a low-density lipoprotein cholesterol level ≥3.62 mmol/L (140 mg/dL), high-density lipoprotein cholesterol level <1.03 mmol/L (40 mg/dL), triglyceride level ≥1.69 mmol/L (150 mg/dL), or lipid-lowering drug use [31]. Diabetes mellitus was defined as a random blood glucose level ≥200 mg/dL, fasting blood glucose level ≥126 mg/dL, hemoglobin A1c level ≥6.5%, or antidiabetic medication use [31]. Body mass index (BMI) was calculated as weight (kg) divided by the height squared (m²).

Estimated glomerular filtration rate (eGFR, mL/min/1.73 m^2^) was calculated using the Japanese equation formula as follows: eGFR = 194 × Cre^–1.094^ × Age^–0.287^ for males, and eGFR = 0.734 × 194 × Cre^–1.094^ × Age^–0.287^ for females [31]. Electrocardiographic left ventricular hypertrophy was defined as Sokolow–Lyon voltage (S wave in V1 + R wave in V5 or V6 ≥ 35 mm) ≥3.5 mV [32]. As only 25 (5.7%) participants met the criteria for left ventricular hypertrophy, the Sokolow–Lyon voltage was used as a continuous variable.

Home BP was defined as self-measured BP at home in the morning using an Omron HEM-747ICN or HEM-7080IC cuff-oscillometric device (Omron Healthcare, Kyoto, Japan) [25, 33]. The participants were instructed to measure their home morning BP levels for 4 weeks, after ≥2 min of rest in the morning within 1 h of waking, before breakfast or taking medications, and keeping the arm-cuff position at heart level during rest, after urination [34]. The first BP measurement in each measurement session was recorded to avoid individual selection bias [34]. Home morning BP was defined as the average of all measurements. Office BP was measured by study staff twice consecutively at each visit using the oscillometric Omron HEM-907IT device or HEM-9000AI (Omron Healthcare Co., Ltd.) [35].

### Statistical analysis

Participants were divided into three groups according to the tertiles of the urinary Na/K ratio (mmol/mmol). Since BNP does not follow a normal distribution, natural log-transformed (ln) BNP values were used in the analysis. To examine the trends of characteristics across the urinary Na/K ratio groups, we used linear regression analysis for normally distributed continuous variables, the Jonckheere–Terpstra test for skewed continuous variables, and the Cochran–Armitage test for categorical variables.

Analysis of covariance with the Tukey–Kramer test for multiple comparisons was employed to assess differences in values across tertiles of the urinary Na/K ratio groups and to calculate adjusted means and standard errors of lnBNP. We then concurrently presented back-transformed BNP values from lnBNP for clinical interpretation. Trend *P-*values were calculated using multiple linear regression models. Covariate adjustments were performed using four sequential models. Model 1 included both sex and age. Model 2 further included BMI, ex-/current smoking and alcohol consumption, dyslipidemia, diabetes mellitus, eGFR, and urine sampling season. Model 3 additionally included home systolic BP, and Model 4 included Sokolow–Lyon voltage as a continuous variable. The adjusted prevalence ratio of BNP ≥ 35 pg/mL across tertiles of the urinary Na/K ratio was estimated using a Poisson regression model with robust variance correction [36].

To compare the associations of the urinary Na/K ratio, estimated 24-h urinary Na, and estimated 24-h urinary K (derived from spot urine) with lnBNP, standardized regression coefficients (β) were calculated by dividing both dependent and independent variables by their respective standard deviation (SD) values.

For sensitivity analyses, stratified analyses were conducted based on sex, age, BMI, ex-/current smoking and alcohol consumption, dyslipidemia, diabetes mellitus, eGFR, home hypertension (systolic BP ≥ 135 mmHg or diastolic BP ≥ 85 mmHg [31]), office hypertension (systolic BP ≥ 140 mmHg or diastolic BP ≥ 90 mmHg [31]), Sokolow–Lyon voltage, and urinary Na/K ratio. Interaction effects between these stratification factors and the urinary Na/K ratio on lnBNP levels were evaluated by incorporating interaction terms into the multiple regression models.

To assess the improvement in discriminative performance when adding the urinary Na/K ratio to the model with covariates, we calculated the change in C-statistic, testing statistical significance using DeLong’s method [37]. Furthermore, we quantified the incremental value of the new variable using the Integrated Discrimination Improvement (IDI) and continuous Net Reclassification Improvement (NRI) metrics for assessing the improvement in average sensitivity and specificity, as well as the correct directional reclassification of individual risk predictions, respectively [38, 39].

Statistical significance was set at an α-level of <0.05 for two-tailed tests. All statistical analyses were performed using SAS version 9.4 (SAS Institute, Cary, NC, USA). Data are expressed as the mean ± SD unless otherwise noted.

## Results

### Participants’ baseline characteristics

Of the 436 participants, 319 (73.2%) were women. The mean age, BMI, and urinary Na/K ratio were 65.4 ± 6.9 years, 23.4 ± 3.2 kg/m², and 3.00 ± 1.52 mmol/mmol, respectively. The mean estimated 24-h urinary Na and K excretions were 168.7 ± 36.2 mmol/day (3877.9 ± 832.7 mg/day, equivalent to 9.8 ± 2.1 g/day of salt) and 46.4 ± 8.8 mmol/day (1814.6 ± 343.7 mg/day), respectively. The median BNP value was 18.6 pg/mL (interquartile range: 11.4–31.2 pg/mL). The group with higher urinary Na/K ratios exhibited a lower prevalence of diabetes mellitus and higher eGFR and home diastolic BP (Table 1).

**Table 1:** Characteristics of participants stratified by urinary Na/K ratio

Characteristics	Tertiles of the urinary Na/K ratio			
	T1: ≤2.19 (n = 145)	T2: 2.19–3.27 (n = 145)	T3: ≥3.28 (n = 146)	Trend *P*
Age, years	65.6 ± 6.6	66.0 ± 7.1	64.8 ± 6.8	0.31
Women, %	77.2	73.8	68.5	0.092
BMI, kg/m^2^	23.4 ± 3.1	23.6 ± 3.4	23.2 ± 3.0	0.57
Ex-/Current smoker, %	16.6	11.0	21.9	0.22
Ex-/Current drinker, %	40.0	37.9	43.2	0.58
Dyslipidemia, %	55.9	46.9	48.6	0.22
Diabetes mellitus, %	26.9	15.9	10.3	0.0002
eGFR, mL/min/1.73 m^2^	75.6 ± 13.7	79.2 ± 12.2	80.1 ± 14.8	0.0047
Home systolic BP, mmHg	124.8 ± 12.3	126.1 ± 14.3	127.5 ± 12.4	0.078
Home diastolic BP, mmHg	74.0 ± 7.7	74.4 ± 9.0	76.2 ± 7.9	0.021
Sokolow-Lyon voltage, mV	2.3 ± 0.7	2.3 ± 0.7	2.4 ± 0.7	0.79
Urinary Na/K ratio, mmol per mmol	1.6 ± 0.4	2.7 ± 0.3	4.7 ± 1.4	<0.0001
Estimated 24-hUNaE, mmol/day	143.2 ± 28.6	170.1 ± 27.4	192.6 ± 33.8	<0.0001
[Salt equivalent, g/day]	[8.4 ± 1.7]	[9.9 ± 1.6]	[11.2 ± 2.0]	<0.0001
Urinary Na/Cre, mmol/g	139.1 ± 63.5	215.4 ± 81.8	297.8 ± 124.5	<0.0001
Estimated 24-hUKE, mmol/day	49.0 ± 8.8	47.1 ± 8.2	43.2 ± 8.3	<0.0001
Urinary K/Cre, mmol/g	86.1 ± 31.4	79.7 ± 27.9	65.4 ± 26.4	<0.0001
BNP, pg/mL*	17.3 (10.2–27.1)	19.1 (12.1–31.3)	21.3 (12.4–36.0)	0.025

^*^Median (interquartile) is shown

*BMI* body mass index, *BNP* brain natriuretic peptide, *BP* blood pressure, *eGFR* estimated glomerular filtration rate, *Na/K* sodium-to-potassium, *24-hUNa (or K) E* 24-h urinary sodium (or potassium) excretion, *T* tertile

The cutoff value of 2.19 mmol/mmol appears in both T1 and T2 due to rounding at the tertile boundary

### Association between urinary Na/K ratio and lnBNP

Table 2 shows the lnBNP levels across tertiles of the urinary Na/K ratio. The highest lnBNP level was observed in the top tertile of the urinary Na/K ratio. The association between the urinary Na/K ratio and lnBNP remained significant after adjusting for sex, age, BMI, ex-/current smoking and alcohol consumption, dyslipidemia, diabetes mellitus, eGFR, urine sampling season (summer, winter, or other), home systolic BP, and Sokolow–Lyon voltage. A significant correlation was observed between the urinary Na/K ratio and lnBNP (*r* = 0.17, Supplementary Fig. 1).

**Table 2:** Adjusted BNP values by urinary Na/K ratio tertiles

	lnBNP (standard errors) [BNP values*]				
Model	T1: Urinary Na/K ratio ≤2.19 (n = 145)	T2: Urinary Na/K ratio of 2.19–3.27 (n = 145)	T3: Urinary Na/K ratio ≥3.28 (n = 146)	ANCOVA *P*	Trend *P*
Model 1	2.77 (0.06) [15.91 pg/mL]	2.89 (0.06) [18.00 pg/mL]	3.03 (0.06)† [20.61 pg/mL]	0.011	0.0026
Model 2	2.74 (0.06) [15.48 pg/mL]	2.88 (0.06) [17.76 pg/mL]	3.07 (0.06)† [21.46 pg/mL]	0.0016	0.0004
Model 3	2.75 (0.06) [15.57 pg/mL]	2.88 (0.06) [17.79 pg/mL]	3.06 (0.06)† [21.31 pg/mL]	0.0028	0.0006
Model 4	2.74 (0.06) [15.50 pg/mL]	2.88 (0.06) [17.81 pg/mL]	3.06 (0.06)† [21.37 pg/mL]	0.0023	0.0005

Model 1 included sex and age

Model 2 added body mass index, ex-/current smoking and ex-/current alcohol consumption, dyslipidemia, diabetes mellitus, estimated glomerular filtration rate, and the season of urine sampling (summer, winter, or others) to Model 1

Model 3 further included home morning systolic blood pressure in addition to the covariates in Model 2

Model 4 additionally included Sokolow-Lyon voltage (as a continuous variable) to the covariates in Model 3

*BNP* brain natriuretic peptide, *ANCOVA* analysis of covariance, *Na/K* sodium-to-potassium

*BNP values were calculated as exponential transformation of the corresponding lnBNP estimation

†*P* < 0.05 vs Tertile (T) 1 group after Tukey-Kramer adjustment

The urinary Na/K ratio, higher estimated 24-h urinary Na excretion, higher urinary Na/Cre ratio, lower estimated 24-h urinary K excretion, and urinary K/Cre ratio were associated with lnBNP levels (Table 3). Among these, the absolute standardized regression coefficient (β) value, representing lnBNP per 1-SD increase in the independent variable, was highest for the urinary Na/K ratio ( | 0.24 | ), followed by urinary Na excretion indices ( | 0.16| for estimated 24-h urinary Na and |0.17| for urinary Na/Cre ratio) (Model 4 in Table 2).

**Table 3:** Regression analysis of the association between urinary Na/K ratio, Na, or K excretions and lnBNP

Independent variable	Model	β	*P*
Urinary Na/K ratio	Model 1	0.19	<0.0001
(1 SD = 1.52 mmol/mmol)	Model 2	0.24	<0.0001
	Model 3	0.23	<0.0001
	Model 4	0.24	<0.0001
24-hUNaE	Model 1	0.08	0.10
(1 SD = 36.2 mmol/day)	Model 2	0.15	0.0041
	Model 3	0.15	0.0041
	Model 4	0.16	0.0029
Urinary Na/Cre	Model 1	0.11	0.020
(1 SD = 113.7 mmol/g Cre)	Model 2	0.16	0.0015
	Model 3	0.16	0.0019
	Model 4	0.17	0.0014
24-hUKE	Model 1	–0.13	0.0078
(1 SD = 8.8 mmol/day)	Model 2	–0.10	0.047
	Model 3	–0.09	0.061
	Model 4	–0.09	0.070
Urinary K/Cre	Model 1	–0.11	0.019
(1 SD = 29.9 mmol/g Cre)	Model 2	–0.10	0.049
	Model 3	–0.10	0.054
	Model 4	–0.09	0.061

Standardized regression coefficient (β) values represent the relationship between each variable and lnBNP after dividing both the independent and dependent variables by their respective 1-SD values

The 1-SD value for lnBNP was 0.76. Standard errors for all estimates were 0.05

Models are adjusted with the same covariates as described in Table 2

*BNP* brain natriuretic peptide, *Na/K* sodium-to-potassium, *24-hUNa (or K) E* 24-h urinary sodium (or potassium) excretion, *Na (or K)/Cre* sodium (or potassium) -to-creatinine, *SD* standard deviation

**Fig. 2:**
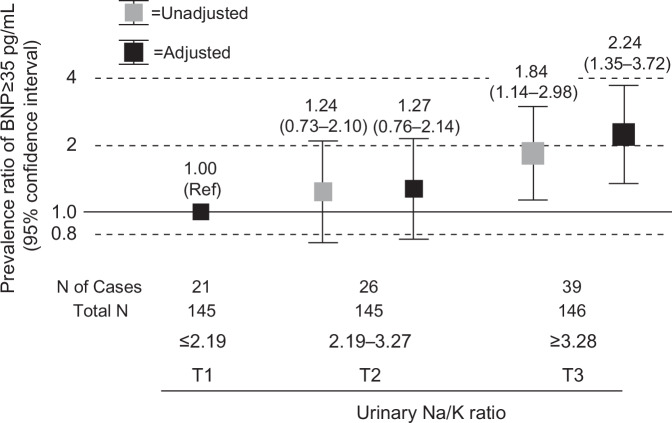
Prevalence ratios (95% confidence intervals) of BNP ≥ 35 pg/mL according to Urinary Na/K Ratio. In the adjusted model, covariates were adjusted for sex, age, body mass index, ex-/current smoking and alcohol consumption, dyslipidemia, diabetes mellitus, estimated glomerular filtration rate, urine sampling season (summer, winter, or other), home morning systolic blood pressure, and Sokolow–Lyon voltage (Model 4 in Table 2). BNP, brain natriuretic peptide; Na/K ratio, sodium to potassium ratio

There were 86 participants (19.7%) with BNP levels ≥35 pg/mL. The adjusted prevalence ratio for BNP ≥ 35 pg/mL increased progressively across tertiles of urinary Na/K ratio (Fig. 2).

### Sensitivity analyses

After replacing home BP with office BP in the adjusted models, the associations between urinary Na/K ratio and ln BNP or BNP ≥ 35 pg/mL remained unchanged (Supplementary Table 1). We stratified the data based on sex, age ( <65/ ≥ 65 years), BMI ( <25/ ≥ 25 kg/m²), ex- or current smoking and alcohol consumption, dyslipidemia, diabetes mellitus, eGFR ( <60/ ≥ 60 mL/min/1.73 m²), home hypertension, office hypertension, Sokolow–Lyon voltage ( <3.5/ ≥ 3.5 mV), and urinary Na/K ratio ( <4/ ≥ 4 mmol/mmol). The analysis revealed no significant interactions between these variables and lnBNP in the urinary Na/K ratio (interaction, *P* ≥ 0.15; Table 4).

**Table 4:** Association between urinary Na/K and lnBNP according to patient characteristics

Variable	Strata	n	β (Standard Errors)	*P*	Interaction *P*
Age	<65 years	219	0.24 (0.07)	0.0011	0.71
	≥65 years	217	0.25 (0.07)	0.0002	
Sex	Women	319	0.24 (0.06)	<0.0001	0.66
	Men	117	0.27 (0.11)	0.014	
BMI	<25 kg/m^2^	311	0.23 (0.06)	<0.0001	0.37
	≥25 kg/m^2^	125	0.29 (0.11)	0.0089	
Smoking	None	364	0.22 (0.05)	<0.0001	0.99
	Ex-/Current	72	0.35 (0.15)	0.023	
Alcohol consumption	None	260	0.25 (0.07)	0.0001	0.72
	Ex-/Current	176	0.23 (0.08)	0.0033	
Dyslipidemia	Absent	216	0.23 (0.06)	0.0002	0.58
	Present	220	0.25 (0.08)	0.0022	
Diabetes mellitus	Absent	359	0.23 (0.05)	<0.0001	0.55
	Present	77	0.23 (0.15)	0.12	
eGFR	<60 mL/min/1.73 m^2^	34	0.30 (0.42)	0.49	0.92
	≥60 mL/min/1.73 m^2^	402	0.24 (0.05)	<0.0001	
eGFR	60–89 mL/min/1.73 m^2^	323	0.24 (0.05)	<0.0001	0.86
( ≥ 60 mL/min/1.73 m^2^)	≥90 mL/min/1.73 m^2^	79	0.23 (0.12)	0.053	
Home hypertension	Absent	315	0.21 (0.06)	0.0002	0.44
	Present	121	0.27 (0.10)	0.0078	
Office hypertension	Absent	290	0.19 (0.06)	0.0041	0.15
	Present	146	0.33 (0.08)	<0.0001	
Sokolow–Lyon’s voltage	<3.5 mV	411	0.25 (0.05)	<0.0001	0.64
	≥3.5 mV	25	−0.03 (0.24)	0.90	
Urinary Na/K ratio	<4 mmol per mmol	350	0.27 (0.10)	0.0058	>0.99
	≥4 mmol per mmol	86	0.28 (0.14)	0.048	

Standardized regression coefficient (β) values indicate the associations between the urinary Na/K ratio and lnBNP, after dividing these values by their respective 1-standard deviations (1.52 and 0.76, respectively). The results were adjusted for sex, age, BMI, ex-/current smoking and alcohol consumption, dyslipidemia, diabetes mellitus, eGFR, season of urine sampling (summer/winter), home morning systolic blood pressure, and Sokolow–Lyon voltage (corresponding to Model 4 in Table 2). *Na/K* sodium-to-potassium ratio, *BNP* brain natriuretic peptide, *BMI* body mass index, *eGFR* estimated glomerular filtration rate

When the urinary Na/K ratio was added to the model containing all covariates (Model 4 in Table 2) for predicting BNP ≥ 35 pg/mL, the C-statistics increased from 0.7043 to 0.7308; however, this change was not statistically significant (0.026, 95% confidence interval [CI]: –0.0046 to 0.058, *P* = 0.095). However, both the IDI (0.036, 95% CI: 0.018–0.054, *P* = 0.0041) and continuous NRI (0.14, 95% CI: 0.013–0.27, *P* = 0.031) showed modest but statistically significant improvements in prediction.

## Discussion

The urinary Na/K ratio was associated with lnBNP levels and remained statistically significant even after adjusting for covariates, including home systolic BP and Sokolow–Lyon voltage. Based on standardized regression coefficients, the urinary Na/K ratio exhibited a stronger and more distinct association than the estimated 24-h urinary Na or K excretion alone. Participants in the highest tertile of the urinary Na/K ratio had an approximately 2.2-fold higher prevalence ratio for BNP levels ≥35 pg/mL compared to the lowest tertile.

This study demonstrated, for the first time, a significant association between the urinary Na/K ratio and BNP levels in a general population. Participants with an apparent history of cardiovascular diseases were excluded. Although the urinary Na/K ratio derived from spot urine has recently been linked to atrial fibrillation [40], participants with atrial fibrillation were also excluded from this study. These findings suggest that the urinary Na/K ratio may be associated with subclinical heart disease. The urinary Na/K ratio can be easily measured in spot urine samples during medical and health checkups. This measurement could help clinicians provide dietary recommendations to optimize Na and K intake for heart failure prevention.

The urinary Na/K ratio, calculated from spot urine samples, could serve as a useful index for evaluating the balance between Na and K intake related to organ damage. This is because the urinary Na/K ratio was more strongly associated with lnBNP levels than the estimated 24-h urinary Na or K excretion. Previous Japanese studies have reported associations between estimated 24-h urinary Na excretion and BNP levels [19–21]. However, a study of 2961 Dutch people (aged 59.8 ± 8.2 years, 48.1% women) indicated that low 24-h urinary K excretion was associated with high N-terminal (NT)-proBNP level, whereas 24-h urinary Na excretion showed no such association [41]. The variability of 24-h urinary electrolyte excretion estimated from spot urine has also been reported [42]. Further studies are necessary to confirm the reproducibility and external validity of the association between urinary Na excretion and BNP levels.

The association between high salt intake and elevated BNP may be explained by inflammation, increased left ventricular volume, left ventricular hypertrophy, or elevated BP [24]. Our study demonstrated that the urinary Na/K ratio was associated with lnBNP levels, independent of electrocardiographic index for left ventricular hypertrophy and elevated BP. Therefore, inflammation and an increase in left ventricular volume without the presence of left ventricular hypertrophy may represent potential mechanisms linking the urinary Na/K ratio to lnBNP. The Dietary Approaches to Stop Hypertension diet, characterized by high consumption of fruits, vegetables, and low-fat dairy products, has been associated with lower urinary Na/K ratios [43] and reduced NT-proBNP levels in multiple studies [44, 45]. These findings suggest that understanding the trend of the urinary Na/K ratio as an indicator of a healthy diet may help prevent BNP elevation.

### Limitations

This study has some limitations. First, the cross-sectional design prevented the establishment of causal relationships. The elevated urinary Na/K ratio could have been a consequence of increased BNP levels, as BNP can promote Na excretion through the urine [24]. Furthermore, it should be noted that the observed association between Na/K ratio and BNP might be specific to measurements taken from samples collected on the same day. Second, our study relied on single spot urine measurements, which may affect the accuracy of estimated Na and K excretion. Multiple random urine samples are required to accurately estimate the 24-h urinary Na/K ratio [4, 46]. Improvement in measurement methods may be required to establish the urinary Na/K ratio as a predictor for pre-heart failure. However, the urinary Na/K ratio, calculated as a concentration ratio, probably demonstrates better reproducibility than absolute estimated excretion values. Third, the lack of dietary information and echocardiographic data limited the consideration of potential confounding factors and assessment of heart failure severity. Fourth, the mean urinary Na/K ratio in our study population was lower than that reported in other general populations, implying that our participants were more health-conscious [4, 5, 47, 48]. A positive association between urinary Na/K ratio and BNP was consistently observed regardless of whether the urinary Na/K ratio was above or below 4 mmol/mmol. This suggests that our findings have external validity across a wide range of urine Na/K ratios. Finally, while a statistically significant association between the urinary Na/K ratio and BNP levels was observed, it is important to acknowledge that this association was relatively modest. The difference in BNP levels between the lowest and highest tertiles of the urinary Na/K ratio was approximately 5.9 pg/mL. However, even a slight difference at the individual level could have a substantial impact on the population level. Given that the urinary Na/K ratio is an established risk factor for hypertension, the conclusion that urinary Na/K ratio is a crucial index remains unchanged.

### Perspective of Asia

Low potassium intake combined with high sodium intake represents a critical health issue across Asian countries [49]. Specifically in Japan, dietary sodium intake remains high partly due to traditional cultural dietary practices. Our findings suggest that the urinary Na/K ratio can be valuable for monitoring and guiding dietary modifications in Asian populations, where established dietary patterns often lead to sodium-potassium imbalances. Given the rising prevalence of heart failure across Asia, characterized as a “pandemic” [15], our findings may offer a practical tool for early prevention strategies that could help curb this growing burden.

## Conclusions

In conclusion, the urinary Na/K ratio was associated with elevated BNP levels rather than with estimated 24-h Na or K excretion alone in the general population without antihypertensive treatment and cardiovascular disease history. The urinary Na/K ratio, readily measured in urine, may be valuable for early prevention of organ damage and cardiac burden. The Japanese Society of Hypertension Working Group recently recommended a urinary Na/K ratio of 4 mmol/mmol as a feasible target and 2 mmol/mmol as the optimal target [4]. Progressive achievement of these targets may contribute to a reduction in BNP levels. However, future prospective studies incorporating detailed dietary data and echocardiographic findings are needed to establish causality and further validate these associations.

## Supplementary information

Supplementary Material
