# Supplementary material for: Association between urinary sodium-to-potassium ratio and BNP in a general population without antihypertensive treatment and cardiovascular diseases: the Ohasama study
Source: Hypertens Res. 2025 Jun 27;48(9):2292–302. doi: 10.1038/s41440-025-02266-0 (PMC12411227; doi:10.1038/s41440-025-02266-0)
Supplement: Supplementary file 1 — Supplementary Material [file 41440_2025_2266_MOESM1_ESM.pdf]

Supplemental Material

Supplemental to:

Association between Urinary Sodium-to-Potassium Ratio and BNP in a General Population without Antihypertensive Treatment and Cardiovascular Diseases: The Ohasama Study

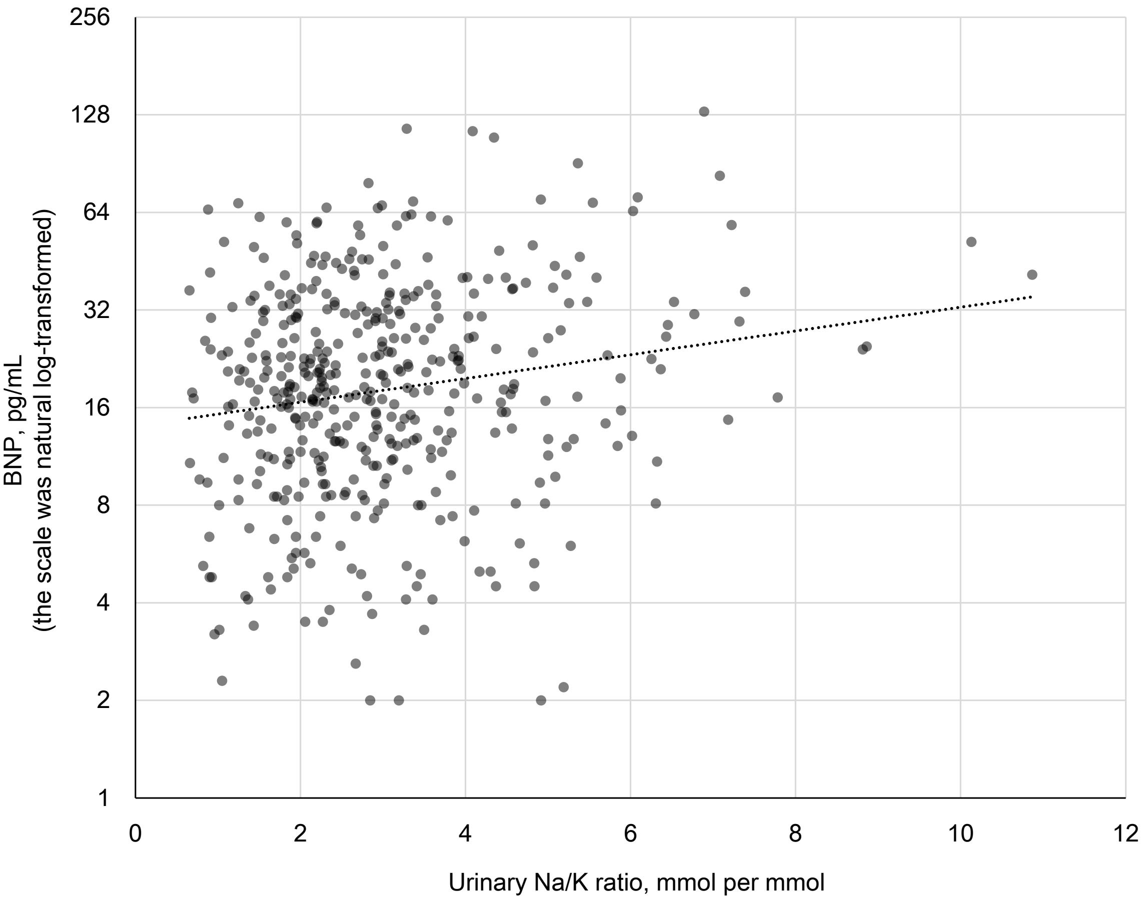

Supplementary Figure 1. Scatter plot of urinary Na/K ratio versus lnBNP

The Pearson's correlation coefficient between urinary Na/K ratio and ln BNP is 0.17.

Na/K: Sodium-to-potassium, ln BNP: Natural logarithm of brain natriuretic peptide

Supplementary Table 1. BNP values and prevalence ratio of BNP≥ 35 pg/mL by urinary Na/K ratio tertiles after adjustment for office systolic blood pressure

Parameters	Tertiles of Urinary Na/K Ratio			Trend *P*
	T1: ≤2.19 (n=145)	T2: 2.19–3.27 (n=145)	T3: ≥3.28 (n=146)	
lnBNP (Standard Errors) [BNP values*]	2.73 (0.06) [15.40 pg/mL]	2.88 (0.06) [17.78 pg/mL]	3.05 (0.06) † [21.22 pg/mL]	0.0005 ‡
Prevalence ratio of BNP≥ 35 pg/mL (95% confidence intervals)	1.00 (Ref)	1.24 (0.74–2.10)	2.18 (1.31–3.64)	0.0024

The results were adjusted for sex, age, body mass index, ex-/current smoking and ex-/current alcohol consumption, dyslipidemia, diabetes mellitus, estimated glomerular filtration rate, and the season of urine sampling (summer, winter, or others), Sokolow-Lyon voltage (as a continuous variable), and office systolic blood pressure.

BNP, brain natriuretic peptide; Na/K, sodium-to-potassium

* BNP values were calculated as the exponential transformation of the corresponding lnBNP estimation.

† *P*<0.05 vs Tertile (T) 1 group after Tukey-Kramer adjustment in the analysis of covariance model.

‡The *P*-value from the analysis of covariance was 0.0024 in the analysis with lnBNP as a dependent variable.
